# Concordance Between Life Satisfaction and Six Elements of Well-Being Among Respondents to a Health Assessment Survey, HealthPartners Employees, Minnesota, 2011

**DOI:** 10.5888/pcd13.160309

**Published:** 2016-12-22

**Authors:** Nicolaas P. Pronk, Thomas E. Kottke, Marcia Lowry, Abigail S. Katz, Jason M. Gallagher, Susan M. Knudson, Sachin J. Rauri, Juliana O. Tillema

**Affiliations:** 1 HealthPartners, Minneapolis, Minnesota; 2 HealthPartners Institute, Minneapolis, Minnesota; 3 Harvard T.H. Chan School of Public Health, Boston, Massachusetts; 4 University of Minnesota School of Medicine, Minneapolis, Minnesota.

## Abstract

**Introduction:**

We assessed and tracked perceptions of well-being among employees of member companies of HealthPartners, a nonprofit health care provider and health insurance company in Bloomington, Minnesota. The objective of our study was to determine the concordance between self-reported life satisfaction and a construct of subjective well-being that comprised 6 elements of well-being: emotional and mental health, social and interpersonal status, financial status, career status, physical health, and community support.

**Methods:**

We analyzed responses of 23,268 employees (of 37,982 invitees) from 6 HealthPartners companies who completed a health assessment in 2011. We compared respondents’ answers to the question, “How satisfied are you with your life?” with their indicators of well-being where “high life satisfaction” was defined as a rating of 9 or 10 on a scale of 0 (lowest) to 10 (highest) and “high level of well-being” was defined as a rating of 9 or 10 for 5 or 6 of the 6 indicators of well-being.

**Result:**

We found a correlation between self-reported life satisfaction and the number of well-being elements scored as high (9 or 10) (*r* = 0.62, *P* < .001); 73.6% of the respondents were concordant (high on both or high on neither). Although 82.9% of respondents with high overall well-being indicated high life satisfaction, only 34.7% of those indicating high life satisfaction reported high overall well-being.

**Conclusion:**

The correlation between self-reported life satisfaction and our well-being measure was strong, and members who met our criterion of high overall well-being were likely to report high life satisfaction. However, many respondents who reported high life satisfaction did not meet our criterion for high overall well-being, which suggests that either they adapted to negative life circumstances or that our well-being measure did not identify their sources of life satisfaction.

## Introduction

In 1994, Evans and Stoddart showed that most people seek well-being in their lives, not just good health (1). Subjective well-being is more global than the construct of good health and less prone to fluctuation than the concept of happiness. Well-being comprises an emotional response level of satisfaction with multiple domains including good health and satisfaction with how one’s life is progressing (2). As part of a research program on the determinants of well-being, Gallup Healthways interviewed people in more than 150 countries (3,4). That research found that people’s level of satisfaction with 5 elements of well-being (career status, social status, financial status, physical health, and community support) differentiate between people who are thriving and those who are suffering. Although Gallup’s knowledge base on well-being is probably greater than that of any other organization, both their data and their algorithms are proprietary and not readily available to researchers.

HealthPartners, a nonprofit member-governed integrated health system, provides health care and health insurance services for its members and customers. Located in Bloomington, Minnesota, its mission is to improve the health and well-being of its members, patients, and community. HealthPartners began administering employee health assessments in 1995 and began its current program to improve our members’ well-being in 2008. In the process of developing a strategy to accomplish this, we reviewed literature on well-being and positive psychology, analyzed health assessment data from our members, and held extensive internal discussions. On the basis of this process, HealthPartners adopted a definition of subjective well-being that is similar to that of the Gallup-Healthways organization but is specific to the HealthPartners culture, is anchored by a sense of meaning and purpose, and comprises 6 well-being elements instead of Gallup’s 5: emotional and mental health, social and interpersonal satisfaction, financial status, career status, physical health, and, community support. Despite same or similar element names, we expected that the questions used by the 2 organizations to assess each of the elements of subjective well-being might differ substantively. The objective of our study was to determine the relationship between self-reported life satisfaction with HealthPartners’ 6 elements of well-being.

## Methods

We used data from our 2011 health assessment to develop operational definitions of our 6 well-being elements. We also developed a strategy to measure and track our members’ health and well-being with summary measures (5). Because our survey was brief, we used our data on self-reported life satisfaction as a surrogate measure of the more complex construct of wellbeing. We did not use an extensive set of questions to collect data on each well-being element because our experience is that the length of a survey is a powerful determinant of the response rate: when completion of a survey is not tied to a health benefit incentive, members are unlikely to respond if the survey is more than 2 pages long. Therefore, we sought to determine the extent to which the response to a single question, “How satisfied are you with your life?” was associated with our measure of well-being and discriminated between people with high levels of overall well-being and those without. Data for this cross-sectional analysis come from the responses of 23,268 employees of six companies (out of 37,982 invitees) who completed a health assessment in 2011. Among questions on other subjects, the survey contained the question, “How satisfied are you with your life?” and questions relating to HealthPartners’ 6 well-being elements.

We calculated the number and percentage of respondents reporting high levels of well-being for each of the 6 elements. We also calculated the correlation between the number of well-being elements scored as high and self-reported life satisfaction. Finally, we calculated the concordance between life satisfaction when defined as high (rating it 9 or 10) versus not high (selecting a value of 8 or lower), and the number of well-being elements scored as high. The HealthPartners institutional review board reviewed and approved the study protocol.

## Results

Of the 37,982 employees contacted, 23,268 completed the health assessment yielding a 61.3% response rate. Nearly two-thirds were women, more than three-quarters were aged 30 to 59, and two-thirds had at least some college education (Table 1). Nearly 50% were professionals or managers; 87.0% self-reported as being non-Hispanic white, 4.4% as black, and 3.7% as Asian (Table 1).

**Table 1:** Characteristics of Respondents to a Health Assessment Survey (N = 23,268), HealthPartners^a^ Employees, 2011

Characteristic	N (%)
**Sex**	
Women	14,579 (62.7)
Men	8,689 (37.3)
**Age, y**	
18–29	2,328 (10.0)
30–39	5,440 (23.4)
40–49	6,529 (28.0)
50–59	6,769 (29.1)
60–64	1,750 (7.5)
≥65	452 (2.0)
**Education**	
≤8th grade	46 (0.2)
Some high school	157 (0.7)
High school diploma or GED	2,035 (8.8)
Technical training or associate degree	4,733 (20.3)
Some college	3,292 (14.2)
Bachelor’s degree	8,349 (35.8)
Postgraduate studies	4,656 (20.0)
**Occupation**	
Administrative or support	2,254 (9.7)
Labor or production	1,137 (4.9)
Professional or management	11,552 (49.6)
Sales	755 (3.2)
Service	1,442 (6.2)
Skilled craft	880 (3.8)
Student	74 (0.3)
Technician	2,422 (10.4)
Retired	18 (0.1)
Other	2,734 (11.8)
**Race/ethnicity**	
American Indian or Alaska Native	104 (0.5)
Asian	864 (3.7)
African American	1,013 (4.4)
Hispanic	349 (1.5)
Non-Hispanic white	20,276 (87.0)
Other	103 (0.4)
No answer	559 (2.5)

Abbreviation: GED, general education development.

a Survey of employees of 6 member companies of HealthPartners.

Nearly two-thirds of the respondents met the criteria for physical well-being (Table 2). Only 21.9% met the criterion for financial well-being, 24.0% for career well-being, 60.0% for emotional and mental well-being, 41.9% for community well-being, and 56.2% for social and interpersonal well-being (Table 2).

**Table 2:** Respondents to a Health Assessment Survey (N = 23,268) Reporting High Levels of Elements of Well-Being, HealthPartners^a^, 2011

Well-Being Element	N (%)
Physical	14,738 (63.3)
Emotional and mental	13,961 (60.0)
Career	5,589 (24.0)
Financial	5,101 (21.9)
Community	9,759 (41.9)
Social and interpersonal	13,079 (56.2)

a Survey of employees of 6 member companies of HealthPartners. We defined a high level of well-being for each indicator as a rating of 9 or 10 on a scale of 0 to 10. And we defined an overall level of well-being as a rating of 9 or 10 for 5 or 6 of the 6 indicators of well-being.

Pearson’s coefficient of correlation between the number of well-being elements that we scored high for an individual and the score that each individual selected to describe his or her level of life satisfaction is *r* = 0.62 (*P* < .001) (Table 3). When we define a high level of overall well-being as meeting well-being criteria for 5 or more elements and a high level of life satisfaction as a rating of 9 or 10, 73.6% of the respondents are concordant for both well-being and life satisfaction being high or both not being high. Although 82.9% of respondents with high overall well-being had high life satisfaction, only 34.7% with high life satisfaction met the criteria for high overall well-being.

**Table 3:** Ratings of Well-Being by Respondents to a Health Assessment Survey (N = 23,268), HealthPartners Employees^a^, 2011

No. of Well-Being Elements Rated As High^c^	No. Participants per Rating of Life Satisfaction^b^					
	≤ 6	7	8	9	10	Total
0	1,155	727	333	48	12	2,275
1	1,126	1,319	1,207	277	81	4,010
2	596	1,222	2,022	758	243	4,841
3	243	661	2,002	1,348	609	4,863
4	77	252	1,183	1,284	910	3,706
5	21	62	454	724	1,055	2,316
6	0	9	62	223	963	1,257
Total	3218	4,252	7,263	4,662	3,873	23,268

a Survey of employees of 6 member companies of HealthPartners.

b Scored on a scale of 0 to 10 where 0 = not at all satisfied and 10 = extremely satisfied.

c High level of well-being was defined as rating an element as 9 or 10 on a scale of 0 to 10 and doing so for 5 or 6 of the 6 elements.

## Discussion

In our analysis of the association between self-reported life satisfaction and our construct of well-being, we found that the 2 constructs were highly correlated: when we defined high life satisfaction as selecting a 9 or 10 on a scale of 0 to 10 and high overall well-being as meeting 5 or more of the 6 elements, we classified 73.6% of the survey respondents correctly as either having both high life satisfaction and high well-being or having neither. Although we also found that more than 80% of respondents whom we classified as having high well-being self-reported high levels of life satisfaction, the converse was not true. Two-thirds of those who reported high life satisfaction did not meet our criteria for having high overall well-being. This suggests that they either accommodated to unfavorable life circumstances or that we failed to identify their sources of life satisfaction. Because of the simplicity of the question, “How satisfied are you with your life,” we don’t believe that the lack of concordance between self-reported life satisfaction and our classification for this group is due to misinterpretation of the question. The question is valid on its face, and the association with our well-being measure demonstrates that it also has concurrent validity.

Changes in society, the environment, or individual circumstances influence how people report life satisfaction. For example, one study reported that almost 10% of people changed an average of 3 points on a 10-point scale from the first year to the last 5 years of a 17-year study. In that same study, height, weight, body mass index, and blood pressure were actually more stable than life satisfaction. In addition, life satisfaction appeared to be sensitive to differences in health-related quality of life, health risk behaviors, and chronic conditions (6). Research also shows that higher life satisfaction is associated with as much as 44% fewer doctor visits and a wide range of sociodemographic, psychosocial, and health-related covariates (7).

That we identified a group of people who indicate that they are highly satisfied with life but lack the indicators of high overall well-being does not surprise us. It is well-documented that a significant minority of individuals — about one-third in some cohorts — see opportunity in the same circumstances in which the majority sense only threat or loss (8–10).

Our findings may not be generalizable to all populations. Our study subjects were employed in large companies and were mostly middle-aged, highly trained non-Hispanic whites. Particular care must be taken when attempting to assess minority populations’ life satisfaction and indicators of well-being, particularly when some members of those populations are not native English speakers. However, our analysis does demonstrate that the concordance between life satisfaction and well-being is strong. We believe that, at a population level, self-reported life satisfaction is a measure of overall well-being.
